# Use of Magnetic Folate-Dextran-Retinoic Acid Micelles for Dual Targeting of Doxorubicin in Breast Cancer

**DOI:** 10.1155/2013/680712

**Published:** 2013-12-05

**Authors:** J. Varshosaz, H. Sadeghi-aliabadi, S. Ghasemi, B. Behdadfar

**Affiliations:** ^1^Department of Pharmaceutics, School of Pharmacy and Novel Drug Delivery Systems Research Centre, Isfahan University of Medical Sciences, Isfahan 81745-359, Iran; ^2^Department of Biotechnology, School of Pharmacy, Isfahan University of Medical Sciences, Isfahan 81745-359, Iran; ^3^Department of Materials Engineering, Isfahan University of Technology, Isfahan 84156-83111, Iran

## Abstract

Amphiphilic copolymer of folate-conjugated dextran/retinoic acid (FA/DEX-RA) was self-assembled into micelles by direct dissolution method. Magnetic iron oxide nanoparticles (MNPs) coated with oleic acid (OA) were prepared by hydrothermal method and encapsulated within the micelles. Doxorubicin HCl was loaded in the magnetic micelles. The characteristics of the magnetic micelles were determined by Fourier transform infrared (FT-IR) spectroscopy, thermogravimetric analysis (TGA), transmission electron microscopy (TEM), and vibrating sample magnetometer (VSM). The crystalline state of OA-coated MNPs and their heat capacity were analyzed by X-ray diffraction (XRD) and differential scanning calorimetry (DSC) methods, respectively. The iron content of magnetic micelles was determined using inductively coupled plasma optical emission spectrometry (ICP-OES). Bovine serum albumin (BSA) was used to test the protein binding of magnetic micelles. The cytotoxicity of doxorubicin loaded magnetic micelles was studied on MCF-7 and MDA-MB-468 cells using MTT assay and their quantitative cellular uptake by fluorimetry method. TEM results showed the MNPs in the hydrophobic core of the micelles. TGA results confirmed the presence of OA and FA/DEX-RA copolymer on the surface of MNPs and micelles, respectively. The magnetic micelles showed no significant protein bonding and reduced the IC_50_ of the drug to about 10 times lower than the free drug.

## 1. Introduction

Breast cancer is the most frequently diagnosed cancer in women that ranks second as the cause of cancer death after lung cancer and as a result there are a large number of studies performed to find novel and effective treatments. Presently combination of chemotherapeutic agents has been developed for breast cancer therapy [1–3].

Doxorubicin is among the most active anticancer agents widely used in treatment of solid tumors and leukemia [1] with cytotoxic, cytostatic, and antineoplastic effects. It works by attacking cells that grow quickly such as cancer cells, but due to its no specificity in inducing cell death some side effects such as bone marrow depression, reduced immunity, cardiovascular toxicity, and several side effects are arisen [4, 5]. Since then, much research has been done for targeting this drug to decrease its side effects, increase its toxic dose in targeted tissues, and deliver the drug exclusively [6–10].

One strategy for improving the antitumor selectivity and toxicity profile of cytotoxic agent is use of magnetic carriers [11–15]. Superparamagnetic iron oxide nanoparticles (Fe_3_O_4_) with a core ranging from 10 nm to 100 nm in diameter are powerful targeted delivery vehicles in various biomedical applications [12]. These particles have organic or inorganic coating, on or within which a drug may be loaded and they are delivered by an external magnetic field to their target tissue. Furthermore, when the external magnetic field is removed they do not exhibit any residual magnetic interaction at room temperature and hence they are unlikely to agglomerate and so uptake by phagocytes. Therefore, they remain in the circulation after injection and pass through the capillary systems of organs and tissues avoiding vessel embolism and thrombosis [13, 16]. Some of these nanoparticles coated by synthetic and natural polymers or stabilized in micro- and nanogels, colloidal systems, liposomes, micelles, and microcapsules or transferred by cationic lipids, polylysine, and protamine sulfate have low entrapment efficiency of drug molecules, release drug molecules immediately not at the appropriate site, or make the particle size larger than the desirable range. Therefore, they do not show enough stability and have tendency to aggregate which leads to toxicity [17]. Such formulations are predominantly taken up by phagocytes of the reticuloendothelial system and cleared from blood circulation before they are able to reach the site of the tumor cells and finally reduce the magnetic nanoparticles efficiency [18]. Recently various anticancer drugs including paclitaxel, methotrexate, mitoxantrone, and doxorubicin have been conjugated with magnetic nanoparticles to enhance tumor targeting [15, 19–21].

There are a number of suitable methods for drug delivery in nanoparticulate systems such as physical complex with hydrophobic chemotherapeutic drug [15, 22, 23] and cleavable covalent linkage [21] that release the drug molecule at the target site. Drugs loaded by hydrophobic interactions typically attach to the surface of magnetic nanoparticles and limit unspecific cellular interaction. This approach provides solutions to drugs that interact with healthy tissue. There are several methods for drug targeting; it can be achieved either by passive targeting or by active targeting [18]. Conjugating the therapeutic molecules such as methotrexate that has affinity to target cells on the surface of magnetic nanoparticles is helpful, but in other cases the use of physical interactions such as electrostatic interactions and hydrophilic/hydrophobic interactions is more useful for coupling of drug molecules on the surface of magnetic nanoparticles.

There are some reports that use magnetic nanoparticles for delivery of chemotherapeutic agents. Hence, they should be capable not only for drug delivery but also for protecting the loaded drug. As a result, coating and loading methods are essential to release drug in appropriate site and at a desired rate. Superparamagnetic iron oxide loaded into polymeric micelles assembled from a copolymer of polyethylene glycol and poly-*ε*-caprolactone has been used for increasing MRI sensitivity [24]. Magnetic nanoparticles modified by 3-aminopropyltrimethoxysilane have been reported for methotrexate delivery and MRI imaging. These magnetic nanoparticles are capable of targeting folate receptors in MCF-7 cell line in breast cancer [21].

Due to the overexpression of the folate receptors in a number of malignant cell lines such as ovary, breast, brain, lung, and colorectal cancers [25, 26] and low expression of the folate receptor in a number of normal human tissues [27] in the present study we used this targeting agent that is overexpressed at breast cancer tissues of the type of MCF-7 to internalize the designed drug delivery system through endocytosis. Hence MCF-7 cells were used as the cell lines that express folate receptors more than MDA-MB-468 cells to comprise the effect of folic acid targeting on internalization of optimal formulation in breast cancer.

For this purpose micelle forming FA/DEX-RA copolymer was used to coat the magnetic nanoparticles and load doxorubicin to target the breast cancer cells dually. This copolymer provides micelles with a core architecture wherein the hydrophobic core served as a carrier for hydrophobic magnetic nanoparticle coated with oleic acid, while the hydrophilic shell interacted with doxorubicin HCl as a hydrophilic drug.

Previous studies on FA/DEX-RA showed that this copolymer was nontoxic and had high capacity to produce micelles enabling the particle stabilization in aqueous solutions [7]. The aim of this study was production of a dual targeted drug delivery system of doxorubicin for breast cancer treatment which could reduce the side effects of the drug in nontargeted tissues while accumulating it in the cancerous locations. For this purpose, FA/DEX-RA was used as the coating biomaterial for the magnetic nanoparticles which was loaded with doxorubicin and further application of an external magnetic field could localize them in target tissues and internalize them within the desired cells.

## 2. Materials and Methods

### 2.1. Materials

Iron (II) chloride tetra hydrate (FeCl_2_·4H_2_O) 99%, oleic acid, bovine serum albumin (BSA), dimethyl sulfoxide (DMSO) anhydrous grade, and Triton X100 were purchased from Merck Chemical Company (Germany). Sodium hydroxide was purchased from PanreaQuimica Co., S.L.U. (Barcelona, Spain). Doxorubicin HCl was provided from Hangzhou ICH Biopharm Co., Ltd. (Zhejiang, China). Deionized water freshly purged with nitrogen gas was used in all steps of the synthesis of magnetic nanoparticles and preparing all aqueous solutions. Dulbecco's phosphate buffered saline (PBS), 0.25% trypsin, and 3-[4,5-dimethylthiazol-2-yl]-2, 5-diphenyl tetrazolium bromide (MTT) were from Sigma (USA). MCF-7 and MDA-MB-468 human breast cancer cell lines were obtained from the Pasteur Institute of Iran. RPMI-1640 medium was from PAA, Austria and penicillin-streptomycin mixtures 50 IU/cc from GIBCO Laboratories, Scotland.

All the chemicals and reagents were used without further purification.

### 2.2. Synthesis of Magnetic Nanoparticles

Iron oxide nanoparticles were prepared by hydrothermal method performed in aqueous media in an autoclave made of Teflon with stainless steel cylinder. The pressure of the oven was regulated at higher than 2000 psi at 180°C for 20 h. Fifty milliliters of oleic acid was added to 80 mL of ethanol 96% while stirring on a magnetic stirrer (IKA PT Lopower, Germany). The mixture was stirred for 10 min and then the solution of 3 M sodium hydroxide was added dropwise over 5 min to the mixture of ethanol and oleic acid to adjust the pH on 7.5. Then 10 mg of iron (II) chloride tetra hydrate (FeCl_2_·4H_2_O) was dissolved in 20 mL of deionized water on a magnetic stirrer for 15 min to obtain 1 M Fe (II) solution. Next, the alcoholic solution of oleic acid was mixed with the solution of Fe on continuous stirring. The prepared solution was transferred to the autoclave and heat treated in the oven at 180°C for 20 h. After the hydrothermal reaction, the autoclave was cooled at room temperature. Finally the resulting magnetic precipitate was separated from large aggregates which were not magnetic using a magnet with the strength of 0.420 Tesla and after removing the aggregates the precipitated magnetic particles were washed three times by ethanol 96%. Afterward, the magnetic nanoparticles were resuspended in 20 mL of water and finally lyophilized (Christ, *α*-2-4 LDPlus, Germany) to make them easier to resuspend in water for subsequent uses.

### 2.3. Preparation of Magnetic Micelles of FA/DEX-RA

FA/DEX-RA copolymer was prepared as reported before [7] and used for producing micelles entrapping oleate coated MNPs and doxorubicin. For this purpose 20 or 30 mg of this copolymer was dissolved in 100 mL of deionized water in which 3 mg of doxorubicin HCl and MNPs were added as much as 25–33% of the copolymer weight. Loading of the drug and MNPs into the micelles was done by shaking, heating, and then sonicating the mixture [12]. The mixture was shaken for 0.5–1 h on an orbital shaker (DAIKI Sciences, Republic of Korea) at a rate of 70–150 rpm at the temperature of 40–60°C and then 2 min of bath sonication (Hawashin 505, Republic of Korea).

To evaluate the effect of processing variables on the responses of particle size, loading efficiency (LE%), drug release efficiency (RE%), Fe_3_O_4_ loading efficiency (Fe E%), and screening the most effective ones an irregular factorial design was proposed by Design Expert software (Version 7.1, USA). Five different variables including polymer content (mg/100 mL), Fe_3_O_4_ (percent of the polymer weight), shaking time (h), shaking rate (rpm), and temperature (°C) were studied each in two levels.  Table 1 shows the five control factors selected in the optimization study.

An overview of the investigated formulations is presented in Table 2. In all formulations the drug content was constant (3 mg). A run involved the corresponding combination of levels to which the factors in the experiment were set. All experiments were done in triplicate. The effects of the studied variables on the responses were then analyzed by the Design Expert software to obtain independently the main effects of these factors, followed by the analysis of variance (ANOVA) to determine which factors were statistically significant. The optimum conditions were determined by the optimization method to yield a heightened performance.

### 2.4. Characterization of OA-Coated Magnetic Nanoparticles and Magnetic Micelles

#### 2.4.1. Particle Size

The particle size and distribution of MNPs were determined using Zetasizer (Zetasizer-ZEN 3600 Malvern Instrument Ltd., Worcestershire, UK) based on dynamic light scattering principle technique. For these measurements 5 mg of freeze-dried powder of MNPs was added to 20 mL of deionized water and ultrasonicated for 1 min (Bath sonicator, HW ASH IN505, Republic of Korea). Then particle size of MNPs suspension was measured at room temperature. An average diameter and distribution of particle size was reported from 3 runs. Furthermore, the mean particle size of FA/DEX-RA micelles loaded with MNPs and drug was measured by the same device suitably diluted to measure mean particle size and polydispersity index of the micelles.

#### 2.4.2. Determination of the Iron Content of Magnetic Micelles

The iron content of magnetic micelles was determined by inductively coupled plasma optical emission spectrometry (ICP-OES, PERKIN ELMER-7300 DV, USA) using 10 milliliter of micelle suspensions loaded with MNPs. The analysis of sample was done compared with the ICP-MS standard (Sigma PerkinElmer Iron (Fe) Pure Grade Atomic Spectroscopy Calibration Standard was supplied with a comprehensive Certificate of Analysis that documented the quality and reliability. Concentration; 1,000 mg/L; matrix is 2% HNO_3_, Volume is 500 mL).

#### 2.4.3. Determination of Doxorubicin Loaded in the MNPs Micelles

Drug loading efficiency (LE%) was determined by measuring the concentration of unencapsulated or free drug in aqueous medium. For this purpose 400 *μ*L of the drug-loaded micelles was centrifuged (Microcentrifuge Sigma 30k, UK) at 10000 rpm for 15 min in microcentrifuging filter tubes (Amicon Ultra, Ireland) with a 10 kDa molecular weight cutoff, and the concentration of free drug in the aqueous medium diluted 1 : 10 with deionized water was measured by a UV-visible spectrophotometer (UV-mini 1240, Shimadzu, Kyoto, Japan) at *λ*_max⁡_ = 274 nm. Unloaded micelles were used as control. The amount of entrapped drug was determined through the difference between the total and the free drug. Loading efficiency (LE%) was calculated by the following equation:

(1) Drug  loading  efficiency  % =Drugtotal−DrugfreeDrugtotal×100.

#### 2.4.4. *In Vitro* Release of Doxorubicin from Micelles

The *in vitro* release of doxorubicin from micelles was monitored in phosphate buffered solution (PBS) 0.2 M (pH 7.4) containing 2% of Tween 20. 4 mL of aqueous micellar dispersion of each formulation was placed in the dialysis membrane bags (Mw cutoff 12000, Membra-Cel, Viskase, USA) and the end-sealed dialysis bags were sunk fully in 15 mL of release medium at room temperature. At appropriate time intervals 600 *μ*L samples were taken and the concentration of doxorubicin released in the medium was determined by UV spectrophotometry method at *λ*_max⁡_ = 499.4 nm. The parameter of release efficiency within 3 h (RE_3_%) was used to compare the release profiles:

(2) $$\begin{array}{c} {\text{RE}}_{3}\%=\frac{\int_{0}^{t}y\cdot dt}{y100\cdot t}\times 100. \end{array}$$

#### 2.4.5. Physical Characterization of MNPs and Micelles Loaded with MNPs and Drug

For the physical characterization of OA-coated MNPs the Fourier transform infrared (FT-IR) spectra, X-ray diffraction (XRD), thermogravimetric analyzer (TGA), and differential scanning calorimetric analysis (DSC) were performed. Besides, the physical characterization of optimal formulation of micelles loaded with MNPs and drug was analyzed by FT-IR spectroscopy. To determine the OA and polymer content of the micelles thermogravimetric analysis (TGA) was performed. The FT-IR of particles was recorded employing a FT-IR Spectroscope (JASCO, FT/IR-6300, Japan). Data was acquired in range of 400–4000 cm^−1^. X-ray diffraction (XRD) patterns of nanoparticles were recorded employing an X-ray diffractometer (EXPERT-MPDX PHILLIPS, The Netherlands) using Cu radiation at *λ* = 0.1546 nm and operating at 40 kV and 40 mA. The samples were mounted on double sided silicone tape and measurements were performed at 2*θ* from 20 to 70°. Thermoanalytical technique of nanoparticles was accomplished on a simultaneous Thermal Analysis device (STA) (LINSEIS L81/1750-PLATINUM, Germany) from 21°C to 725°C at a heating ramp of 10°C, under a constant flow of nitrogen gas (100 mL/min). In this regard, thermogravimetric analysis (TGA) and differential scanning calorimetry (DSC) were performed too.

#### 2.4.6. Particle Morphology

The morphology of OA-coated MNPs and also the optimal formulation of micelles loaded with MNPs and drug were evaluated by transmission electron microscope (Zeiss, EM10C, Germany). The sample for TEM measurements were prepared by placing a droplet of the suspension onto a 300 mesh carbon coated copper grid and allowing it to dry in air naturally. Finally, micrographs were taken with different levels of magnification with an accelerating voltage of 80 kV.

#### 2.4.7. Magnetic Properties of Micelles

Magnetic parameters of the prepared MNPs and the micelles loaded with MNPs were measured by a vibrating sample magnetometer (VSM) (AGFM/VSM 3886 Kashan, Iran) at room temperature in a magnetic field strength of 1 Tesla.

### 2.5. Protein Binding Measurements

To determine the protein binding interaction with MNPs after trapping in the micelles 2 and 5 mL of BSA solution (2 mg/30 mL) were added to optimal formulation of micelles loaded with MNPs on separate beakers and the mixtures were shaken for 1 h at 150 rpm at 37°C. After filtering the mixture with a Millipore filter with porosity of 50 nm the UV absorbance of the resulting solution was evaluated by the UV-visible spectrophotometer at *λ*_max⁡_ = 277.5 nm. Finally the results were compared with the total UV absorption of the blank micelles and free BSA solution at the same wavelength.

### 2.6. *In Vitro* Cytotoxicity

MCF-7 (as breast cancer cells overexpressing folate receptors) and MDA-MB-468 cells (as breast cancer cells not expressing folate receptors) were seeded in 96-well plates at 2 × 10^4^ cells/mL and grown for 24 h. The cells were then treated with free doxorubicin, doxorubicin and MNPs loaded in FA/DEX-RA micelles, micelles of FA/DEX-RA loaded with MNPs but doxorubicin-free, and blank FA/DEX-RA micelles without drug and MNPs. All these groups were tested in the absence of magnetic field (0.420 Tesla). Then FA/DEX-RA micelles loaded with doxorubicin and MNPs at different concentrations of 0.05,0.1,0.5, and 1 *μ*M were cultured in the magnetic field at 37°C for 48 h. After this period, each well was exposed to 20 *μ*L of MTT and plates were incubated in a CO_2_ incubator (Napco 6500, French) for an additional 3 h. Then wells of the culture medium were removed and blue-violet formazan crystals were dissolved by adding 150 *μ*L of DMSO. The color intensity was measured at wavelength of 570 nm using an ELISA plate reader (Awareness, USA). Untreated cells and cells treated with doxorubicin were used as negative and positive controls, respectively. Standard deviations were obtained from 3 replicate for each cell line.

### 2.7. Measurement of Cellular Uptake of MNPs Loaded in Micelles

The MCF-7 and MDA-MB-468 breast cancer cell lines were maintained in RPMI 1640 medium supplemented with 10% fetal bovine serum and 1% of antibiotics mixture (penicillin/streptomycin 50 IU/cc) at 37°C in a humidified and 5% CO_2_ atmosphere. Breast cancer cells were seeded in 24-well plates (2×10^5^ cells per well) and allowed to grow for 24 h. The medium was replaced with 1 mL/well of FA/DEX-RA micelles loaded with 1 *μ*M doxorubicin and MNPs and incubated for 2 h at 37°C in both absence and presence of magnetic field (0.420 Tesla). After that the cells were washed three times with PBS and scraped by trypsin (250 *μ*L in each well) then 500 *μ*L of medium was added to each well to neutralize the effect of trypsin. The breast cancer cells were finally lysed by adding 1 : 10 Triton X100 (250 *μ*L) for 10 min at the incubator and the resulting pellets were used to determine cellular uptake by fluorimetry method. Doxorubicin was used as the fluorescent probe marker and calibration curve was constructed by measuring the fluorescence intensities of the solutions with known concentrations of doxorubicin at *λ*_em_ = 550 nm and *λ*_exc_ = 475 nm by a Fluorescence Spectrometer (Perkin Elmer LS-3, USA). Untreated cells were used as blank.

### 2.8. Stability Test of Different Formulations

The particle size was measured every 48 h till 30 days for evaluation of the stability of different formulations of micelles loaded with MNPs.

### 2.9. Statistical Analysis

Values were processed using Microsoft Excel 2010 and IBM SPSS Statistics 20 using analysis of variance (ANOVA) followed by the post hoc test of LSD and the level of significance was set at *P* < 0.05. The effects of the studied variables on the responses in optimization of the formulation of micelles were analyzed by the Design Expert software (Version 7.1, USA) to obtain independently the main effects of studied variables, followed by the ANOVA test to determine which factors were statistically significant and the optimum conditions were determined by the optimization method to yield a heightened performance.

## 3. Results and Discussion

### 3.1. Synthesis of Magnetic Nanoparticles

There are several methods to produce stable superparamagnetic iron oxide nanoparticles [11, 12, 28]. Although most of these methods provide convenient way to synthesize MNPs, but they also face some disadvantages such as large particle size distribution, aggregation, poor crystallinity, complicated purification, large amount of solvent required, and cost of their production and require exhaustive control of experimental conditions [12, 18, 28]. In this study, the hydrothermal method was used to develop MNPs since this method has some advantages like feasibility of large scale production and relative cost effectiveness. Besides there is well control on the agglomeration of metal nanoparticles and the nanoparticles are easily modified for synthesis in the presence of oleic acid and other surfactants [29, 30]. Hence, this method finally results in synthesis of MNPs coated with oleic acid which can be encapsulated into the hydrophobic core of prepared micelles.

### 3.2. Characterization of OA-Coated MNPs and Micelles of FA/DEX-RE Loaded with MNPs and Doxorubicin

**Scheme 1:**
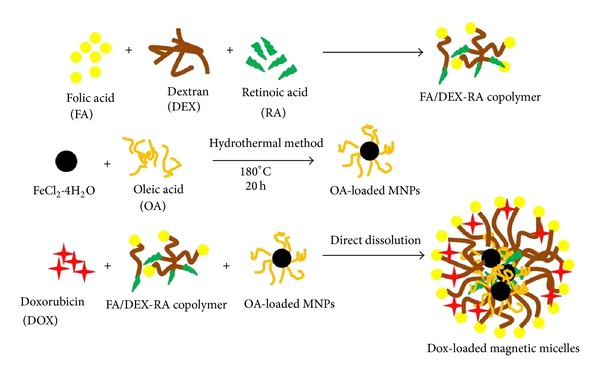
Formation of doxorubicin loaded magnetic micelles.

OA-coated MNPs and anticancer drug of doxorubicin were loaded into the folate targeted micelles, as shown in Scheme 1.

#### 3.2.1. Particle Size and Zeta Potential

The particle size and distribution measurements of MNPs coated with oleic acid were obtained using a dynamic light scattering (DLS) instrument. They showed the mean particle size of 20 nm with a polydispersity index of 0.2 which indicates low diversity of the particle size. The routes of synthesis of superparamagnetic iron oxide nanoparticles play an important role in determination of the particle size, shape, and size distribution [12, 28]. Adjusting parameters such as total concentration of cations, temperature, pressure, and pH can lead to monodisperse particles and desirable size by using hydrothermal method [31, 32]. In this respect, the temperature used was set at 180°C for 20 h which produced nanosized particles with well-defined crystal shape and high homogeneity [24, 33]. Besides, the surface of the iron oxide core was coated with oleic acid which led to minimize the size distribution and aggregation of the magnetic cores in the process of hydrothermal method [34]. The results of physicochemical properties of different formulations of micelles loaded with MNPs and doxorubicin are shown in Table 3.

**Figure 1:**
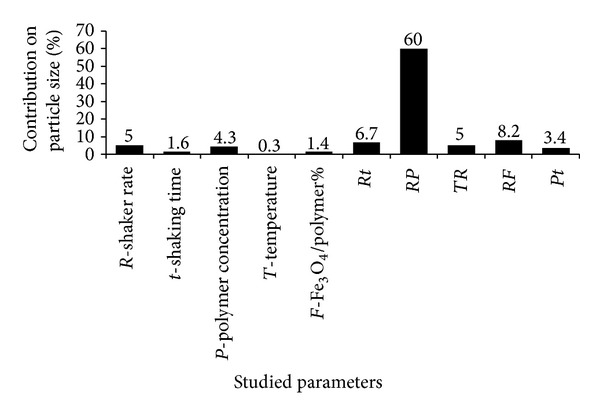
Contribution percentage of different effective factors on the particle size of Dox-loaded magnetic micelles (*Rt* = interaction effect of shaker rate and shaking time, *RP* = interaction effect of shaker rate and polymer concentration, *TR* = interaction effect of temperature and shaker rate, *RF* = interaction effect of shaker rate and Fe_3_O_4_/polymer%, and *Pt* = interaction effect of polymer concentration and shaking time).

As seen polydispersity index (PDI) of the prepared magnetic micelles was between 0.3 and 0.5 (Table 3) which indicates low diversity of the particle size. PDI is calculated from the square of the standard deviation/mean diameter; thus less value of PDI indicates enhanced homogeneity of the magnetic micelles [7]. As it can be seen in Figure 1, the most effective parameter on the particle size of the magnetic micelles was the interaction between shaking rate and polymer content and the other parameters had no significant effect on the particle size. Variety groups of polymers with different concentration have been used to coat MNPs to serve functional goals [11]. Changing the polymer content besides polymer type may affect the particle size and PDI [22]. Furthermore, the particle size could be decreased with increasing the shaker rate [7] due to aggregation of particles in their medium [35, 36]. It is reported that temperature and medium type are two important factors that control the particle size [37, 38], but in the present study the effect of temperature was rare (Figure 1). Our previous studies on the physicochemical properties of FA/DEX-RE copolymer showed that the drug concentration had a significant impact on particle diameter of micelles [6, 7]. However, in the present study the drug content of magnetic micelles was kept constant. After entrapping the MNPs in the copolymeric micelles the particle size grew (Table 3). It can be considered that the polymer content can have important effect on particle size.

#### 3.2.2. ICP Analysis

**Figure 2:**
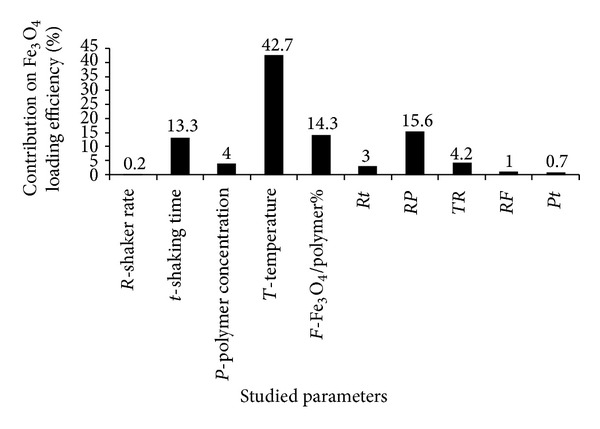
Contribution percentage of different effective factors on the Fe_3_O_4_ loading efficacy% of Dox-loaded magnetic micelles (*Rt* = interaction effect of shaker rate and shaking time, *RP* = interaction effect of shaker rate and polymer concentration, *TR* = interaction effect of temperature and shaker rate, *RF* = interaction effect of shaker rate and Fe_3_O_4_/polymer%, and *Pt* = interaction effect of polymer concentration and shaking time).

The ICP analysis confirmed the entrapment of superparamagnetic iron oxide nanoparticles into micelles (Table 3). As Figure 2 indicates many factors affected the Fe_3_O_4_ loading in nanoparticles such as shaking time, Fe_3_O_4_/polymer ratio, shaking rate, polymer content interaction, and temperature, but as it can be seen in Figure 2 temperature was the most important factor affecting loading efficiency of Fe_3_O_4_, while the role of other factors was negligible and changing temperature from level 1 to level 2, that is, increasing the temperature, decreased the Fe_3_O_4_ loading in micelles due to increasing the particle size and aggregation of OA-coated MNPs. Some studies reported that environmental factors such as temperature have a main effect on shape, zeta potential, particle size, magnetic properties, and morphology [32]. So an appropriate range of temperature is suggested to load magnetic nanoparticles in each particular formulation. Although the increased temperature in current study led to decrease Fe_3_O_4_ loading efficiency but in another study worked on carboxylated polyamidoamine dendrimers it has been shown that elevated temperature caused highly stable and soluble magnetodendrimers [12]. Moreover, previous report indicated that the temperature played an important role in controlling the swelling state of the poly-n-isopropylacrylamide (PNIPAM) as a thermoresponsive polymer which influenced the drug release and loading efficacy [39]. In other words, enhanced loading of the magnetic nanoparticles in some polymers by increasing the temperature may be due to the swelling properties of the carrier polymer in response to the temperature and consequently higher loading of Fe_3_O_4_ is reported. However, in our study no swelling depended on temperature was seen in the copolymeric micelles and hence no extra loading of Fe_3_O_4_ was seen by temperature enhancement, but on contrary it was reduced.

#### 3.2.3. Determination of Doxorubicin Loading in the Magnetic Micelles

**Figure 3:**
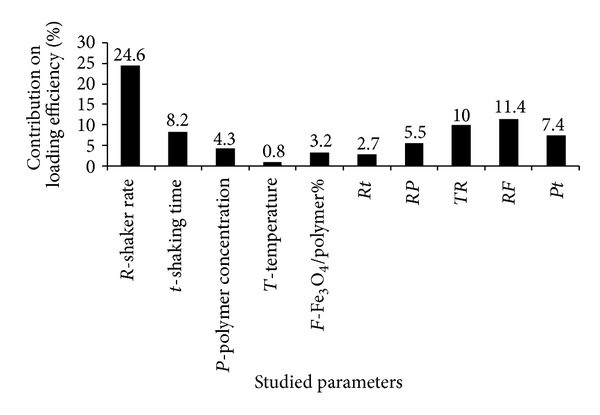
Contribution percentage of different effective factors on loading efficiency% of Dox-loaded magnetic micelles (*Rt* = interaction effect of shaker rate and shaking time, *RP* = interaction effect of shaker rate and polymer concentration, *TR* = interaction effect of temperature and shaker rate, *RF* = interaction effect of shaker rate and Fe_3_O_4_/polymer%, and *Pt* = interaction effect of polymer concentration and shaking time) (*P* is the polymer content of 20 or 30%, *R* is the shaking rate of 75 or 150 rpm, and *t* is the shaking time of 0.5 or 1 h) and *T* represents the temperature of 40 or 60°C).

Considering the studied variables, that is, shaking rate (75–150 rpm), shaking time (0.5–1 h), temperature (40–60°C), and Fe_3_O_4_/copolymer (25–33% of copolymer weight), it can be seen in Figure 3 that the shaker rate was the most important factor affecting the drug loading efficiency. So that by increasing the shaker rate, the loading efficiency decreased effectively. Therefore, it may be concluded that magnetic nanoparticles coated with FA/DEX-RA copolymer which have negative charged functional groups can couple with ionized doxorubicin via electrostatic interaction in an optimized appropriate shaker rate [7, 11, 12]. As seen in Figure 3, the other effective factors on loading efficiency are shaking time and the interactions of shaker rate with FeE% and temperature. These results show that there are different effective factors on the loading of drugs in different carriers that may be generally divided into two categories, that is, (I) environmental factors and (II) the manner in which the drug interacts with the specific polymeric carriers such as polymeric nanoparticles, liposomes, and micelles [12]. Although our previous study [7] showed that increased polymer content resulted in less drug loading efficiency, in the present study the effect of polymer content on this parameter was lower than the environmental factors such as shaking rate and time and their interaction (Figure 3). This might be because the amount of polymer used in this study was nearly in the optimum range like the pervious study. As a result, using FA/DEX-RA copolymer in a desirable range can ensure high loading efficiency. The nature and amount of the drug are the other factors that affected the loading efficiency (Figure 3). Morales et al. [40] reported that the high hydrophobicity of paclitaxel made it difficult to develop into an effective drug delivery system. In the current study we cannot represent the effect of the drug content on the loading efficiency due to a constant concentration of doxorubicin used in all formulations, but obviously, as mentioned before, the positive charge of doxorubicin caused its attachment to the corona of magnetic micelles. However, Yoo and Park [6] reported that doxorubicin loading efficiency in polyethylene glycol-folate conjugate polymer gradually decreased as the initial applied drug was increased. As a result it may be concluded that the environmental factors along with the polymer type and concentration, the way that the drug binds to the polymer, and the nature and amount of the drug play an important role in controlling the drug loading efficiency and lead to success release of the drug molecule at the target site.

#### 3.2.4. *In Vitro* Release of Doxorubicin from Magnetic Micelles

**Figure 4:**
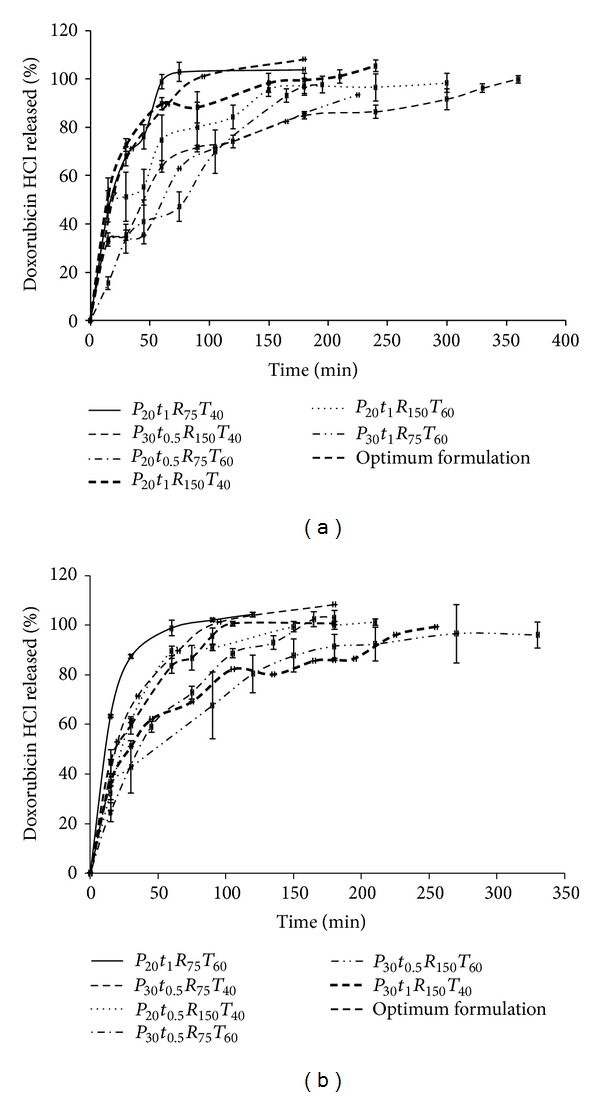
Release profiles of doxorubicin HCl from different studied formulations of FA/DEX-RA magnetic micelles with the ratio of Fe_3_O_4_/polymer (a) 33% and (b) 25% (in all formulation codes *P* is the polymer content of 20 or 30%, *R* is the shaking rate of 75 or 150 rpm, *t* is the shaking time of 0.5 or 1 h, and *T* represents the temperature of 40 or 60°C).

Figure 4 shows the release profiles of doxorubicin versus time for each studied magnetic micellar formulation. This figure indicates that all formulations except for *P*_20_ *F*_25_ *t*_1_ *R*_75_ *T*_60_, *P*_20_ *F*_33_ *t*_1_ *R*_75_ *T*_40_, and *P*_30_ *F*_25_ *t*_0.5_ *R*_75_ *T*_40_ released almost 100% of the loaded drug during 120 min, 75 min, and 105 min, respectively. In the others drug release lasted for more than 3 h. In all of them after a rapid burst release the remaining drug was released with a near zero order kinetic. This may be interpreted as doxorubicin dissolved in the corona of magnetic micelles was leaked promptly into the release medium and the next slow release phase was due to the drug diffusion through the inner layer of the magnetic micelles [19, 41]. In this study the pH of the release medium was considered to be constant by using phosphate buffer solution (PBS) (0.2 M, pH 7.4) containing 2% of Tween 20, but previous studies [18, 41] reported that the release of doxorubicin from magnetic micelles was pH dependent; for example, Yang et al. [42] reported that doxorubicin release at pH 5.0 was much faster than that at pH 7.4 from both formulations containing poly(ethylene glycol) and poly(3-caprolactone) copolymers.

**Figure 5:**
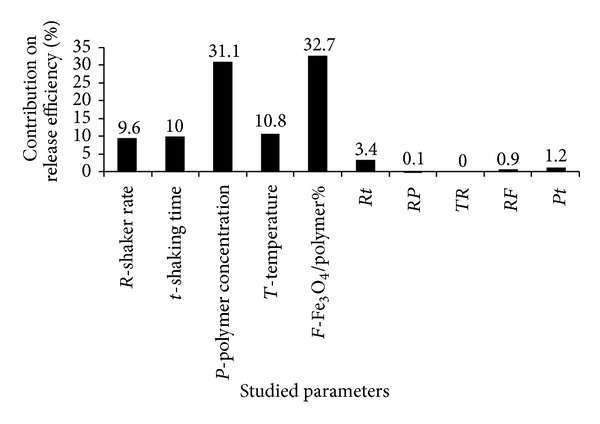
Contribution percentage of different effective factors on release efficiency of Dox-loaded magnetic micelles (*Rt* = interaction effect of shaker rate and shaking time, *RP* = interaction effect of shaker rate and polymer concentration, *TR* = interaction effect of temperature and shaker rate, *RF* = interaction effect of shaker rate and Fe_3_O_4_/polymer%, and *Pt* = interaction effect of polymer concentration and shaking time).

As seen in Figure 5 polymer concentration and Fe_3_O_4_/polymer ratio were two important parameters that affected the release efficiency. Increasing the polymer concentration (i.e., changing from level 1 to level 2) led to decrease the release efficiency possibly by enhanced electrostatic interactions between doxorubicin and the polymer. Hayama et al. [43] reported that in release of camptothecin from polymeric micelles modified by folate-PEG-lipid the higher folate surface density led to the lower drug release due to the affinity interactions of folate coupling. Furthermore, Zhang and Misra [44] demonstrated that an acid-labile hydrazine bond between carbonyl group of doxorubicin and hydrazide group of dextran-g-poly(N-isopropylacrylamide-co-N,N-dimethylacrylamide) affected the release efficiency in acidic environment. As mentioned earlier, the other main effective factor on release efficiency was Fe_3_O_4_/polymer ratio, increasing this ratio decreased the release efficiency from the micelles (Figure 5). Considering this fact that the drug release is governed mainly by two mechanisms of drug molecules diffusion and polymer matrix degradation, it can be concluded that every variable that affects the polymer nature can change the drug release behavior. Zhu et al. [45] represented that increasing the polymer concentration effectively prevented 5-Fu release by forming the thicker polymer wall. Since drug release can be magnetically triggered from the drug-conjugated magnetic nanoparticles [18], it has been shown that at appropriate temperature range and by using thermosensitive polymers with magnetic core drug diffuses out of the micelles under temperature control [14]. Zhang and Misra [44] reported that dextran-g-poly(NIPAAm-co-DMAAm) as a thermosensitive smart polymer can regulate drug release in response to temperature changes by swelling and deswelling in the vicinity of lower critical solution temperature (LCST) ~40°C. However, in our study as Figure 5 indicates that increasing the temperature from level 1 to level 2 decreased the release efficiency and consequently we may conclude that the lower ratio of Fe_3_O_4_/polymer along with the lower level of temperature led to enhanced drug release. Decreasing the temperature in the production process of the micelles that increased the drug release efficiency probably has caused the loose arrangement and less coiling of the hydrophilic dextran blocks of FA/DEX-RA around the core and easier release of the entrapped drug. This effect agrees well with what Nayebsadrian et al. [7] have reported before. Thus, under our experimental conditions, the drug release is influenced by the copolymer content, Fe_3_O_4_/polymer ratio, and temperature (Figure 5). Notably, another aspect for future research is using magnetic field and studying its effect on the controlled release of the trapped drug.

#### 3.2.5. Physical Characterization of Magnetic Nanoparticles and Micelles

**Figure 6:**
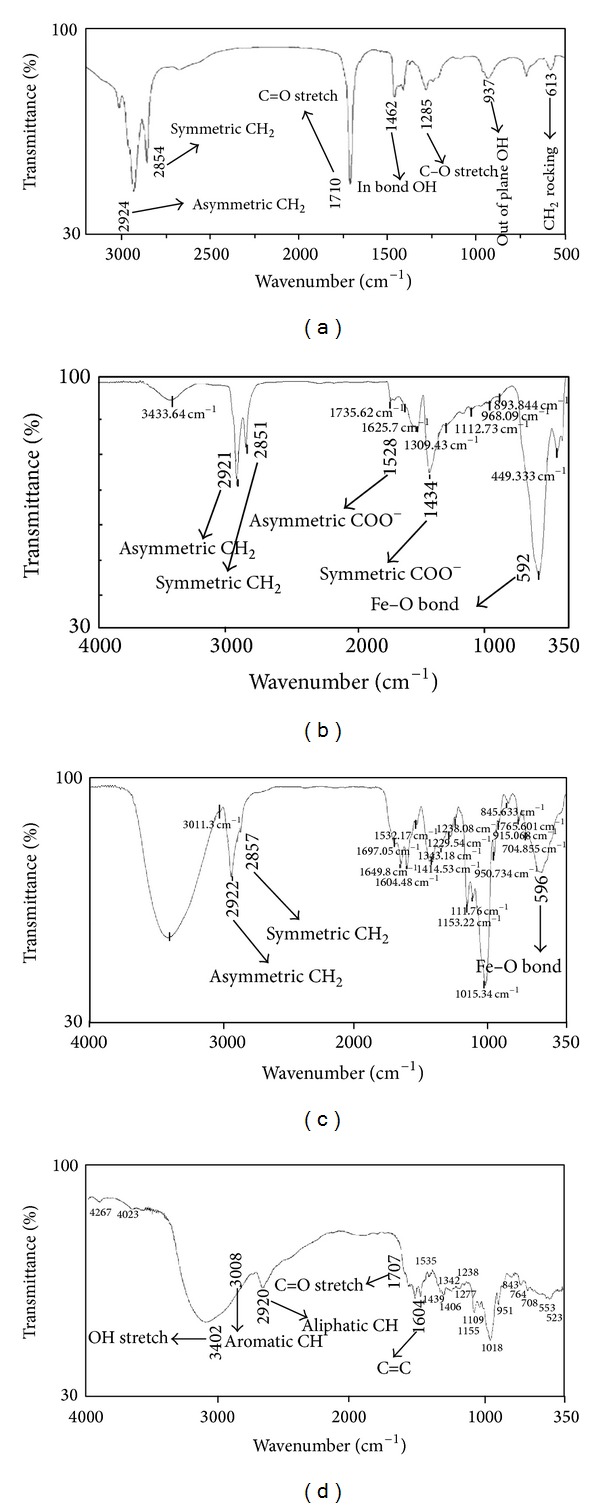
FT-IR spectra: (a) magnetic MNPs, (b) OA-coated magnetic MNPs, (c) FA/DEX-RA coated magnetic micelles, and (d) FA/DEX-RA copolymer.

To confirm the presence of oleic acid on the surface of Fe_3_O_4_ nanoparticles, FT-IR analysis was done (Figure 6(a)). The core of magnetic nanoparticles produced a sharp signal in 592 cm^−1^ due to Fe–O bonds and it is attributed to supramagnetic properties of Fe_3_O_4_ nanoparticles [44, 46, 47]. In this spectrum the asymmetric CH_2_ and the symmetric CH_2_ stretching bonds shifted from 2921 and 2851 cm^−1^, to a lower frequency region, respectively, compared to pure oleic acid (Figure 6(b)). This is because of a crystalline state caused by the monolayer of oleic acid as a hydrocarbon molecule surrounding the magnetic nanoparticles [46]. The adsorption bond around 1700–1730 cm^−1^ in the spectrum of pure oleic acid (Figure 6(b)) is due to the C=O stretching bond of the carboxyl group that is absent in the spectrum of the coated magnetic nanoparticles (Figure 6(a)). Instead, it shows two new bonds at 1434 and 1528 cm^−1^ revealing the asymmetric (COO–) and the symmetric (COO–) stretching that is caused by the bonding of the oleic acid molecules to the magnetic nanoparticle surface [48]. Pure oleic acid exhibited that a broad peak at 2400–3400 cm^−1^ (Figure 6(b)) corresponds to O–H stretching vibration of carboxylic acids [47], but it became much weaker that it is assigned to the chelating bidentate type interaction between the COO– group and the Fe atoms [46].  Figure 6(c) represents the FT-IR spectra of MNPs coated with FA/DEX-RA copolymer. In this spectrum the distinctive peaks are seen at 596 cm^−1^ (characteristic peak of Fe_3_O_4_), 2922 and 2857 cm^−1^ the asymmetric CH_2_ stretching and the symmetric CH_2_ stretching bonds of superparamagnetic iron oxide nanoparticles (Fe_3_O_4_) coated with oleic acid, respectively, and the major peaks for FA/DEX-RA (Figure 6(d)) that were represented in a previous study [7].

**Figure 7:**
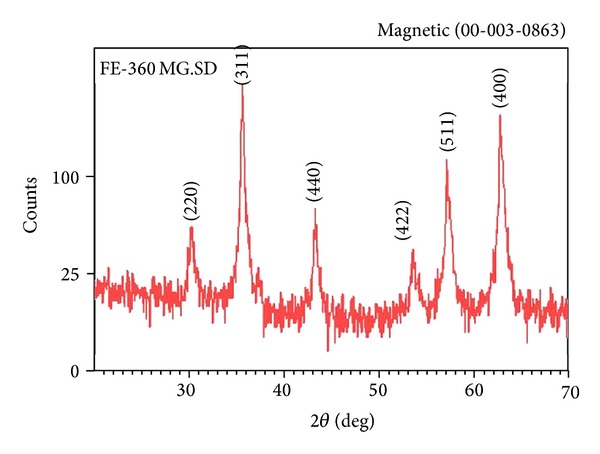
XRD pattern of the magnetite nanoparticles prepared by the hydrothermal method.

Figure 7 shows XRD pattern of the OA-coated MNPs. The crystal size was calculated to be 20.17 nm using Scherrer's equation: Particle  size = 0.9*λ*/*β*cos⁡*θ*, where *λ* is the X-ray wavelength and equal to 0.1546 nanometer, *β* is the line broadening at half the maximum intensity equal to 311, and *θ* is the Bragg angle equal to 35.5/2 = 17.75.

Six characteristics peaks are seen in Figure 7 for Fe_3_O_4_ nanoparticles (2*θ* = 30.1,35.5,43.2,53.4,57.5,62.7) marked by their indices (220,311,400,422,511,440). As can be seen the position and relative intensity of all peaks match well with diffraction data of standard Fe_3_O_4_ powder, indicating that the sample is Fe_3_O_4_ crystal [22, 47].

**Figure 8:**
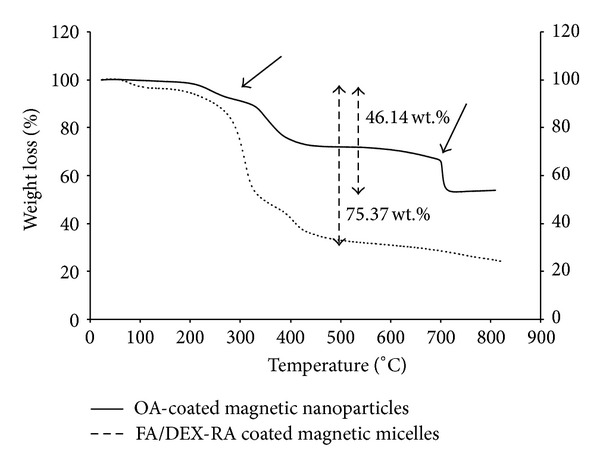
Thermogravimetric analysis of OA-coated magnetic nanoparticles and FA/DEX-RA coated magnetic micelles.

Thermogravimetric analysis was performed to further confirm the presence of oleic acid and FA/DEX-RA copolymer on the surface of the OA-coated MNPs and optimal micelles, respectively. Hence, the freeze-dried powder was used to achieve this analysis. As it can be seen in Figure 8 OA-coated MNPs had a weight loss of ~46.14 wt% (iron oxide core content 53.86 wt%), whereas optimal micelles lost ~75.37 wt% (iron oxide core content 24.63 wt%) and this data agreed with what was reported by ICP method that was used for measuring the loading efficiency of Fe_3_O_4_ in the micelles (Table 3) and can confirm that additional weight loss in the case of optimal micelles is due to FA/DEX-RA coating copolymer. It is clear from the data that the OA-coated MNPs contained nearly 50 wt%. Figure 8 indicates that two desorption processes occurred in the OA-coated MNPs in the vicinity of 210 and 685°C. These different desorption processes were explained by Zhang et al. [46] so that the oleic acid was adsorbed as a bilayer structure on the surface of iron oxide particles. The external layer is expected to be absorbed by physical bonds and the internal layer has a chemically bonding pattern [22].

**Figure 9:**
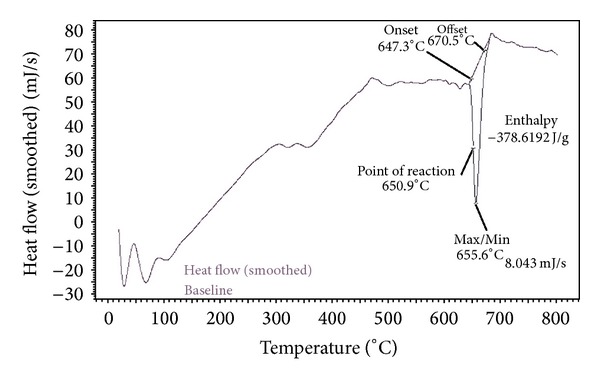
Differential scanning calorimetry (DSC) thermogram of OA-coated magnetic nanoparticles.

Figure 9 shows the DSC thermogram of OA-coated MNPs and indicates the difference in the amount of heat required to increase the temperature of OA-coated MNPs as a function of temperature. A large endothermic transition was found at 655.6°C for OA-coated MNPs, which may be related to the oleic acid molecules binding chemically and directly to the MNPs nanoparticle. Furthermore, the melting point of oleic acid attached physically on the surface of MNPs was observed around 20°C that agrees well with Gonzales and Krishnan's report [49]. This finding is interestingly agreeable with the TGA results seen in Figure 8 that showed that oleic acid was coated on the surface of Fe_3_O_4_ nanoparticles as a bilayer adsorption.

In the study reported by Zhao et al. [50] on characterization of magnetic nanoparticles of Fe_3_O_4_ and CoFe_2_O_4_, they found that the oleic acid molecules were coated as a bilayer adsorption on the surface of Fe_3_O_4_ nanoparticles, while it was a single layer adsorption for CoFe_2_O_4_ nanoparticles.

#### 3.2.6. Particle Size and Morphology

**Figure 10:**
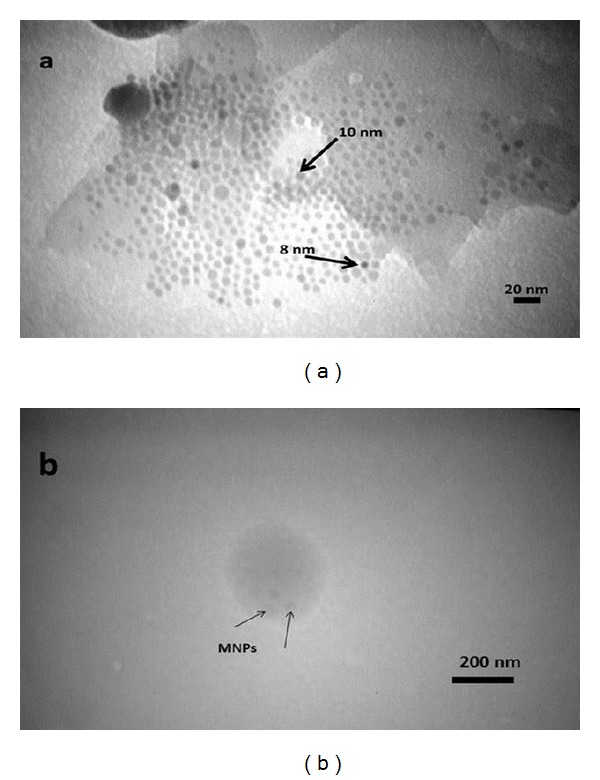
TEM micrographs: (a) OA-coated magnetic MNPs, (b) FA/DEX-RA magnetic micelles.

Particle size and morphology of MNPs can be seen in Figure 10(a) by TEM study. According to the scale bar of the graphs, the results of particle size are in agreement with Scherrer's result discussed before in XRD results. The nanoparticles are obviously discrete spherical shapes and separate from their neighbors by the organic ligands adsorbed on the particles surface. A typical TEM photograph of FA/DEX-RA coated MNPs is shown in Figure 10(b). Results suggest that micelles were fairly smooth and spherical in shape, and the size was ranging between 200 and 250 nm. According to the stability test data (Figure 15), the particle size was expected to shift to 200–300 nm after 24–48 h and it is the reason for much larger particle size obtained from the TEM micrographs compared to DLS method (Table 3). In another aspect, TEM micrograph shows that the Fe_3_O_4_ nanoparticles coated with oleic acid are entered into the micelles core due to their hydrophobic properties [51].

#### 3.2.7. Magnetic Properties

**Figure 11:**
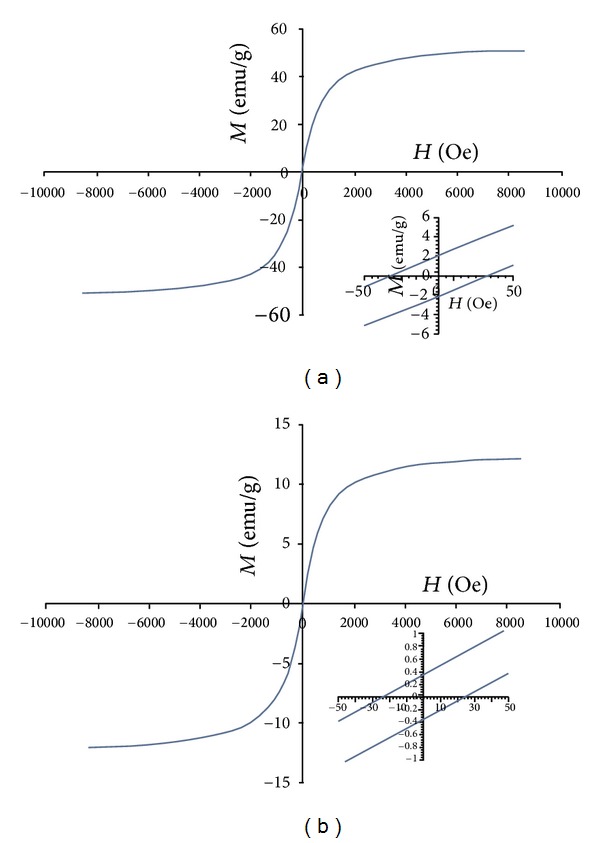
Magnetization curve measured using vibrating sample magnetometer (VSM): (a) OA-coated magnetic MNPs, (b) FA/DEX-RA magnetic micelles.

Figure 11 displays the superparamagnetic property of OA-coated MNPs and FA/DEX-RA magnetic micelles in the powder state at room temperature. The saturation magnetization (*σ*_*s*_) was determined to be 53.6 and 12.58 emu/g for OA-coated MNPs and FA/DEX-RA magnetic micelles, respectively. According to the reduction in *σ*_*s*_ value of magnetic micelles compared to OA-coated MNPs (Figure 11(a)), it can be concluded that the presence of an organic coating on the surface of OA coated iron oxide has led to a decrease in saturation magnetization [35, 51]. The nature of the curves at small magnetic fields shows the coercivity of 26 Oe due to the non-superparamagnetic property at room temperature (Figure 11(b)).

### 3.3. Optimization

Computer optimization process by Design Expert software (version 7.2, USA) and a desirability function determined the effect of the levels of independent variables on the responses. All responses were fitted to the linear model. Table 3 shows that the constraint of particle size was 109.0 ± 4.3 nm ≤ Y1 ≤ 164 ± 7.6 nm with targeting the particle size on minimum, for drug loading efficiency the constraint was 73.0 ± 0.4% ≤ Y3 ≤ 100.0 ± 0.0% with the goal set on maximum, the RE_3_% constraint was 57.4 ± 2.45% ≤ Y4 ≤ 100.2 ± 0.99% with the target set at the range of the obtained results, and for FeE% it was 2.5% ≤ Y5 ≤ 13.5% with the goal set at the maximum. Twelve different formulations were designed with Design Expert software by an irregular factorial design (Table 3) and optimized magnetic micelles processing situation was suggested by desirability of 100% as using FA/DEX-RA copolymer content of 20 mg, Fe_3_O_4_/polymer ratio of 25.4%, temperature of 40.4°C, shaking time of 0.5 h, and shaking rate of 75 rpm. The predicted and actual values of responses are shown in Table 4. These results showed that the error percent was −0.84,0.15,0.02, and −0.04 for particle size, loading efficiency, RE_3_%, and FeE%, respectively. As seen in Table 4 the actual results were in close accordance with the predicted values by the software.

### 3.4. Protein Binding

**Figure 12:**
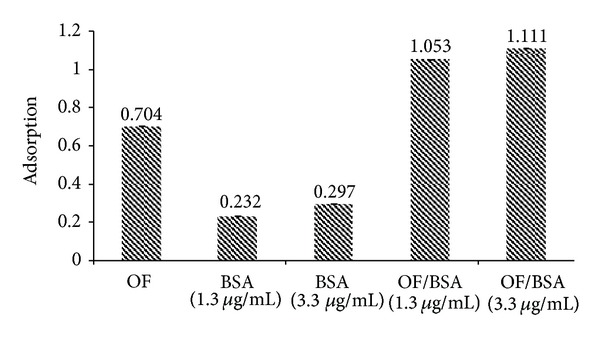
UV absorption of optimal formulation (OF) and bovine serum albumin (BSA) under protein binding test.

The magnetic nanoparticles developed for preclinical setting and human application should be formulated into a suitable delivery system with appropriate pharmacokinetic parameters. Different proteins of the blood serum such as albumin bind to the surface of such particles due to their high hydrophobicity and change their fate before reaching the intended target [52]. In the present study, FA/DEX-RA polymer was used as a biodegradable organic coating [12, 18] to reduce protein adsorption.  Figure 12 shows the total amount of the absorbances of the blank magnetic micelles and the BSA solutions are almost the same as the absorbance of the mixture of these two solutions. The small deviation or increase in the absorbance may be related to the spectrophotometer error. The MNPs demonstrate no significant change in the UV absorbance of the optimal formulation before and after incubation with BSA as an *in vitro* protein binding test. If the protein binding was observed, it was expected that the absorbance to be reduced, but obviously the data indicates that each solution represents its own absorbance at *λ*_max⁡_ = 277.5 nm. In the study reported by Sun et al. [53] on the poly(ethylene glycol) (PEG) coated iron oxide nanoparticles, they found that PEG coating played an essential role to reduce protein adsorption. On the other hand, Yallapu et al. [52] observed a slight change in the surface morphology and particle size of magnetic nanoparticles loaded with curcumin and coated by *β*-cyclodextrin and Pluronic 68 after incubation with mouse or human serum. Although many coating materials can reduce protein adsorption, prolong serum half-life, and thus increase efficiency of the particles internalization by target cells [21, 22, 47], it has been shown that dextran coated particles have completely different adsorption behavior [44]. This makes dextran an appropriate coating material for molecular targeting due to its minimizing of the interaction between the magnetic core and plasma proteins and therefore slower clearance from circulation system [44].

### 3.5. *In Vitro* Cytotoxicity Tests

**Figure 13:**
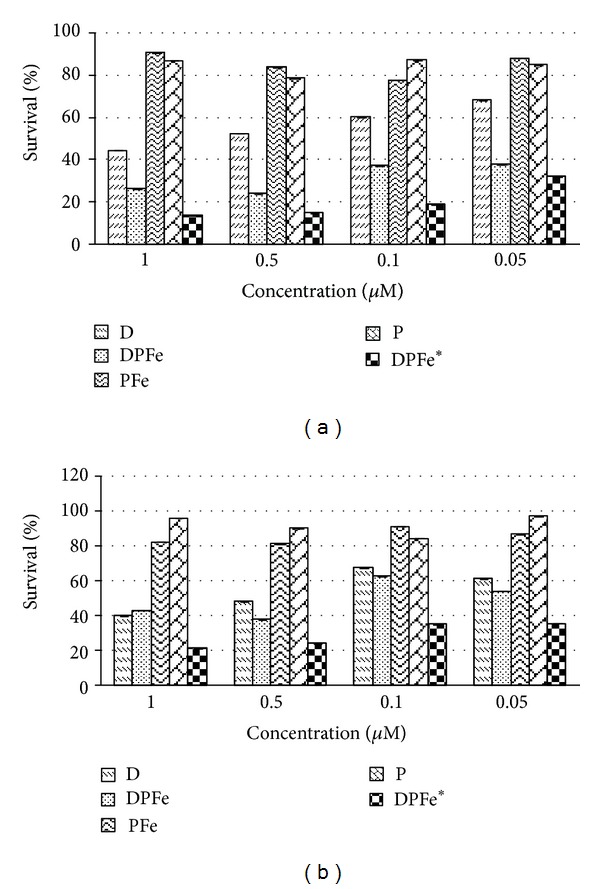
Viability of (a) MCF-7 and (b) MDA-MB-468 cells after treatment with different concentrations of doxorubicin loaded in magnetic micelles of FA/DEX-RA with or without the magnetic field in comparison with free doxorubicin by MTT assay (*n* = 3). D = free doxorubicin, DPFe = Dox-loaded FA/DEX-RA MNPs without magnetic field, PFe = FA/DEX-RA MNPs without Dox, P = FA/DEX-RA copolymer, and DPFe* = Dox-loaded FA/DEX-RA MNPs with magnetic field.

Cell survival percentages of MCF-7 and MDA-MB-468 cells are shown in Figure 13. Doxorubicin loaded magnetic micelles targeted with folic acid in the absence and presence of magnetic field were compared with free doxorubicin. To show the effect of folate targeting, MCF-7 overexpressing folate receptors [20, 42, 54] were compared with MDA-MB-468 cells often considered as negative folate receptor breast cancer cells [54]. As can be seen from Figure 13(a) in MCF-7 cells the survival percentage was decreased in targeted micelles both in absence and presence of magnetic field compared to free doxorubicin. The folate targeted micelles showed the lowest IC_50_ of 0.05 *μ*M compared to free doxorubicin which showed the IC_50_ of 0.5 *μ*M. This effect agreed with what was reported by previous studies and showed that the presence of magnetic external fields besides targeting with special ligands led to increasing cytotoxic effects of different formulations [6, 42, 43, 55].

Figure 13 also shows that the magnetic field led to efficient internalization of magnetic micelles within the cells. So that the highest inhibitory growth effect was observed in cells treated with FA/DEX-RA MNPs in the presence of magnetic field at 1 *μ*M concentration that is approximately two and four times greater than the effect observed with FA/DEX-RA MNPs without local magnetic field and free doxorubicin at the same concentration, respectively.  Figure 13(b) indicates that in MDA-MB-468 cells the most cytotoxic effect occurred at 1 *μ*M concentration of FA/DEX-RA MNPs with applied magnetic field compared to free doxorubicin and FA/DEX-RA MNPs without application of the magnetic field. Interestingly, there were no significant difference between magnetic targeted micelles without magnetic field and free doxorubicin, due to ineffective internalization of FA/DEX-RA MNPs within the folate receptor negative cells (Figure 13(b)). Yang et al. [41] worked on superparamagnetic iron oxide doxorubicin loaded micelles targeted to folic acid receptors and reported that applying an external magnetic field localized the magnetic micelles in KB cells after which folic acid as a targeting ligand would allow their binding to the cell membrane receptors and enhance the micellar uptake inside the tumor cells. In another study, magnetic resonance imaging (MRI) of MCF-7 and L929 cells indicated negative contrast enhancement for MCF-7 cells over L929 cells (folic acid negative receptor) when methotrexate-conjugated magnetic nanoparticles were used [20]. It is noteworthy that as a whole, the dual targeting effect of folate conjugated micelles and loaded Fe_3_O_4_ was more effective in cell growth inhibition because of increasing the concentration of doxorubicin loaded magnetic nanoparticles at the desired site [12].

### 3.6. Cellular Uptake of Magnetic Micelles

**Figure 14:**
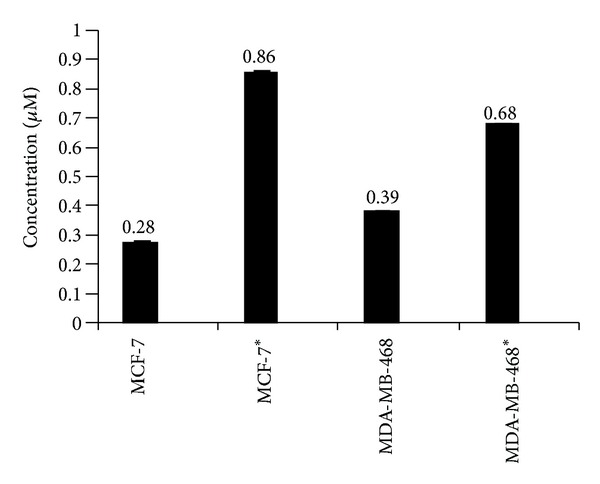
Quantitative estimation of cellular uptake of Dox-loaded FA/DEX-RA MNPs by fluorimetry method at *λ*_em_ = 550 nm and *λ*_ex_ = 475 nm (* = in presence of magnetic field of 0.42 T).

Figure 14 represents the cellular uptake of doxorubicin loaded FA/DEX-RA MNPs in the absence and presence of external magnetic field determined by using fluorimetry method. The quantitative measurement of magnetic micelles uptake by MCF-7 and MDA-MB-468 cells demonstrates higher uptake by MCF-7 cells in the presence of magnetic field. These data are in agreement with results obtained in the MTT assay (Figure 13) and revealed once more the dual targeting effect of Fe_3_O_4_-loaded and folate-conjugated dextran/retinoic acid copolymer to target cells in the presence of a suitable magnetic field. There is significant difference between MCF-7 and MDA-MB-468 in terms of folate targeting (*P* < 0.05). However, further investigation on the several concentrations of folic acid targeted micelles in different magnetic field strengths is necessary to establish the efficacy of the developed magnetic micelles for various therapeutic applications [41].

### 3.7. Stability Test

**Figure 15:**
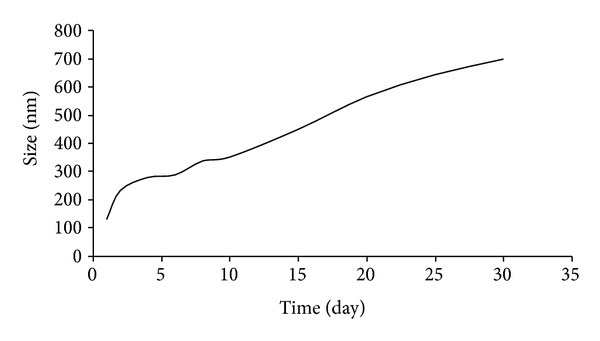
The stability test of the optimized magnetic micelles according to the particle size growth under room temperature.

The stability study was performed by measuring the size of the optimized formulation of magnetic micelles at the various designated time points under room temperature. As it can be seen in Figure 15 that the particle size of the micelles measured by Zetasizer versus time increased with time which is probably due to the aggregation of the micelles with time, but the dispersion was very stable for almost one month. Whereas in the study reported by Chandrasekharan et al. [51] at its best condition the particle size of micelles was just observed to be stable for 12 days. In the current study the optimal formulation showed great stability for one month and neither any aggregation nor sedimentation was observed. Furthermore, these data indicate that the optimal formulation is much stable until 30 days and a gradual increase in particle size from 132 to 698 nm was observed. The results obtained from this test agreed with what was reported by previous studies on dextran micelles [7].

## 4. Conclusions

FA-DEX/RA magnetic micelles were prepared for dual targeted delivery of doxorubicin for breast cancer cells. A micellar carrier from a block copolymer, folate-conjugated dextran/retinoic acid copolymer, has been developed to encapsulate OA-coated magnetic Fe_3_O_4_ and to deliver doxorubicin as the anticancer drug. OA-coated MNPs were synthesized successfully by hydrothermal method. The best formulation of FA-DEX/RA magnetic micelles prepared by direct dissolution method was that containing 20 mg of FA/DEX-RA copolymer, Fe_3_O_4_/polymer ratio of 25.4%, fabrication temperature of 40.4°C, shaking time of 0.5 h, and shaking rate of 75 rpm. The highest growth inhibitory effect was observed in MCF-7 cells treated with FA/DEX-RA MNPs in the presence of magnetic field at 1 *μ*M concentration, so these micelles demonstrated the potential to achieve dual tumor targeting (i.e., magnetic field-guided and folate-targeting) of micelles to breast cancer cells. This may reduce the required dose of doxorubicin and consequently reduces the side effects of this drug. The results should be checked *in vivo* to confirm the promising results obtained from the cell culture tests.

## Figures and Tables

**Table 1:** Description and trial levels of studied factors in irregular factorial design used in preparation of doxorubicin loaded in magnetic micelles of FA/DEX-RA.

Studied variables	Levels	
	I	II
Copolymer content (mg/100 mL)	20	30
MNPs (% of copolymer weight)	25	33
Shaking time (h)	0.5	1
Shaking rate (rpm)	75	150
Temperature (°C)	40	60

**Table 2:** Composition of different formulations investigated in preparation of doxorubicin loaded in magnetic micelles of FA/DEX-RA using irregular factorial design.

Run	Copolymer content (mg/100 mL)	MNPs (% of copolymer weight)	Shaking time (h)	Shaking rate (rpm)	Temperature (°C)
*P*_20_ *F*_25_ *t*_1_ *R*_75_ *T*_60_	20	25	1	75	60
*P*_20_ *F*_33_ *t*_1_ *R*_75_ *T*_40_	20	33	1	75	40
*P*_30_ *F*_25_ *t*_0.5_ *R*_75_ *T*_40_	30	25	0.5	75	40
*P*_30_ *F*_33_ *t*_0.5_ *R*_150_ *T*_40_	30	33	0.5	150	40
*P*_20_ *F*_33_ *t*_0.5_ *R*_75_ *T*_60_	20	33	0.5	75	60
*P*_20_ *F*_25_ *t*_0.5_ *R*_150_ *T*_40_	20	25	0.5	150	40
*P*_30_ *F*_25_ *t*_0.5_ *R*_75_ *T*_60_	30	25	0.5	75	60
*P*_30_ *F*_25_ *t*_0.5_ *R*_150_ *T*_60_	30	25	0.5	150	60
*P*_20_ *F*_33_ *t*_1_ *R*_150_ *T*_40_	20	33	1	150	40
*P*_20_ *F*_33_ *t*_1_ *R*_150_ *T*_60_	20	33	1	150	60
*P*_30_ *F*_33_ *t*_1_ *R*_75_ *T*_60_	30	33	1	75	60
*P*_30_ *F*_25_ *t*_1_ *R*_150_ *T*_40_	30	25	1	150	40

**Table 3:** Particle size, loading efficiency, release efficiency (RE_3_%), and Fe_2_O_3_ loading efficacy % of different doxorubicin loaded in folate-targeted DEX/RA magnetic micelles (mean ± SD; *n* = 3).

Formulation code	Particle size	PDI	Loading efficiency %	RE_3_ %	Fe_3_O_4_ loading efficacy %
*P*_20_ *F*_25_ *t*_1_ *R*_75_ *T*_60_	119.0 ± 4.5	0.4 ± 0.0	100.0 ± 0.0	100.2 ± 1.0	7.0
*P*_20_ *F*_33_ *t*_1_ *R*_75_ *T*_40_	122.0 ± 5.2	0.3 ± 0.0	83.0 ± 0.0	86.9 ± 1.3	9.0
*P*_30_ *F*_25_ *t*_0.5_ *R*_75_ *T*_40_	145.0 ± 8.6	0.4 ± 0.0	92.0 ± 0.5	81.1 ± 1.3	11.0
*P*_30_ *F*_33_ *t*_0.5_ *R*_150_ *T*_40_	117.0 ± 1.7	0.3 ± 0.0	90.0 ± 0.2	61.2 ± 0.9	7.0
*P*_20_ *F*_33_ *t*_0.5_ *R*_75_ *T*_60_	115.0 ± 9.4	0.4 ± 0.1	86.0 ± 0.5	57.4 ± 2.4	3.0
*P*_20_ *F*_25_ *t*_0.5_ *R*_150_ *T*_40_	154.0 ± 9.4	0.3 ± 0.1	91.0 ± 0.4	79.8 ± 0.7	5.0
*P*_30_ *F*_25_ *t*_0.5_ *R*_75_ *T*_60_	146.0 ± 16.3	0.4 ± 0.0	73.0 ± 0.4	71.4 ± 1.0	3.5
*P*_30_ *F*_25_ *t*_0.5_ *R*_150_ *T*_60_	137.0 ± 1.2	0.3 ± 0.0	97.0 ± 0.5	62.9 ± 2.8	6.4
*P*_20_ *F*_33_ *t*_1_ *R*_150_ *T*_40_	135.0 ± 26.4	0.4 ± 0.1	100.0 ± 0.0	81.3 ± 2.0	7.0
*P*_20_ *F*_33_ *t*_1_ *R*_150_ *T*_60_	109.0 ± 4.3	0.5 ± 0.0	100.0 ± 0.0	71.7 ± 3.8	4.1
*P*_30_ *F*_33_ *t*_1_ *R*_75_ *T*_60_	164 ± 7.6	0.4 ± 0.1	86.0 ± 0.4	57.8 ± 2.9	2.5
*P*_30_ *F*_25_ *t*_1_ *R*_150_ *T*_40_	112.0 ± 2.6	0.3 ± 0.0	94.0 ± 0.0	66.4 ± 2.7	13. 5

**Table 4:** Comparison of the Design Expert predicted and actual values of responses studied in Dox-loaded FA/DEX-RA magnetic micelles.

Responses	Size (nm)	Loading efficiency %	RE_3_ %	FeE %
Predicted	83	108	86	13.5
Actual	90.0 ± 0.8	91.0 ± 3.7	84.0 ± 1.4	14
Error%	−0.84	0.15	0.02	−0.04

## References

[1] American Cancer Society (2012). *Breast Cancer Facts and Figures 2011-2012*.

[2] American Cancer Society (2013). *Cancer Facts and Figures 2013*.

[3] Avis NE, Crawford S, Manuel J (2005). Quality of life among younger women with breast cancer. *Journal of Clinical Oncology*.

[4] Wang JJ, Cortes E, Sinks LF, Holland JF (1971). Therapeutic effect and toxicity of adriamycin in patients with neoplastic disease. *Cancer*.

[5] Shi Y, Moon M, Dawood S, McManus B, Liu PP (2011). Mechanisms and management of doxorubicin cardiotoxicity. *Herz*.

[6] Yoo HS, Park TG (2004). Folate-receptor-targeted delivery of doxorubicin nano-aggregates stabilized by doxorubicin-PEG-folate conjugate. *Journal of Controlled Release*.

[7] Nayebsadrian M, Varshosaz J, Hassanzadeh F, Sadeghi H, Banitalebi M, Rostami M (2012). Screening the most effective variables on physical properties of folate-targeted dextran/retinoic acid micelles by taguchi design. *Journal of Nanomater*.

[8] Kratz F, Beyer U, Roth T (1998). Transferrin conjugates of doxorubicin: synthesis, characterization, cellular uptake, and in vitro efficacy. *Journal of Pharmaceutical Sciences*.

[9] Patil R, Portilla-Arias J, Ding H (2012). Cellular delivery of doxorubicin via ph-controlled hydrazone linkage using multifunctional nano vehicle based on poly(*β*-L-malic acid). *International Journal of Molecular Sciences*.

[10] Jhaveri MS, Rait AS, Chung K-N, Trepel JB, Chang EH (2004). Antisense oligonucleotides targeted to the human *α* folate receptor inhibit breast cancer cell growth and sensitize the cells to doxorubicin treatment. *Molecular Cancer Therapeutics*.

[11] Veiseh O, Gunn JW, Zhang M (2010). Design and fabrication of magnetic nanoparticles for targeted drug delivery and imaging. *Advanced Drug Delivery Reviews*.

[12] Wahajuddin DR, Arora S (2012). Superparamagnetic iron oxide nanoparticles: magnetic nanoplatforms as drug carriers. *International Journal of Nanomedicine*.

[13] Lu A-H, Salabas EL, Schuth F (2007). Magnetic nanoparticles: synthesis, protection, functionalization, and application. *Angewandandte Chemie International Edition*.

[14] Chomoucka J, Drbohlavova J, Huska D, Adam V, Kizek R, Hubalek J (2010). Magnetic nanoparticles and targeted drug delivering. *Pharmacological Research*.

[15] Jain TK, Richey J, Strand M, Leslie-Pelecky DL, Flask CA, Labhasetwar V (2008). Magnetic nanoparticles with dual functional properties: drug delivery and magnetic resonance imaging. *Biomaterials*.

[16] Saiyed ZM, Telang SD, Ramchand CN (2003). Application of magnetic techniques in the field of drug discovery and biomedicine. *Biomagnetic Research and Technology*.

[17] Montet-Abou K, Montet X, Weissleder R, Josephson L (2007). Cell internalization of magnetic nanoparticles using transfection agents. *Molecular Imaging*.

[18] Arruebo M, Fernández-Pacheco R, Ibarra MR, Santamaría J (2007). Magnetic nanoparticles for drug delivery. *Nano Today*.

[19] Hu S-H, Tsai C-H, Liao C-F, Liu D-M, Chen S-Y (2008). Controlled rupture of magnetic polyelectrolyte microcapsules for drug delivery. *Langmuir*.

[20] Gao F, Yan Z, Zhou J, Cai Y, Tang J (2012). Methotrexate-conjugated magnetic nanoparticles for thermochemotherapy and magnetic resonance imaging of tumor. *Journal of Nanoparticle Research*.

[21] Kohler N, Sun C, Fichtenholtz A, Gunn J, Fang C, Zhang M (2006). Methotrexate-immobilized poly(ethylene glycol) magnetic nanoparticles for MR imaging and drug delivery. *Small*.

[22] Yallapu MM, Othman SF, Curtis ET, Gupta BK, Jaggi M, Chauhan SC (2011). Multi-functional magnetic nanoparticles for magnetic resonance imaging and cancer therapy. *Biomaterials*.

[23] Guo M, Yan Y, Zhang H (2008). Magnetic and pH-responsive nanocarriers with multilayer core-shell architecture for anticancer drug delivery. *Journal of Materials Chemistry*.

[24] Hong GB, Zhou JX, Yuan RX (2012). Folate-targeted polymeric micelles loaded with ultrasmall superparamagnetic iron oxide: combined small size and high MRI sensitivity. *International Journal of Nanomedicine*.

[25] Garin-Chesa P, Campbell I, Saigo PE, Lewis JL, Old LJ, Rettig WJ (1993). Trophoblast and ovarian cancer antigen LK26: sensitivity and specificity in immunopathology and molecular identification as a folate-binding protein. *American Journal of Pathology*.

[26] Ross JF, Chaudhuri PK, Ratnam M (1994). Differential regulation of folate receptor isoforms in normal and malignant tissues in vivo and in established cell lines. Physiologic and clinical implications. *Cancer*.

[27] Weitman SD, Lark RH, Coney LR (1992). Distribution of the folate receptor GP38 in normal and malignant cell lines and tissues. *Cancer Research*.

[28] Osborne EA, Atkins TM, Gilbert DA, Kauzlarich SM, Liu K, Louie AY (2012). Rapid microwave-assisted synthesis of dextran-coated iron oxide nanoparticles for magnetic resonance imaging. *Nanotechnology*.

[29] Haw CY, Mohamed F, Chia CH (2010). Hydrothermal synthesis of magnetite nanoparticles as MRI contrast agents. *Ceramics International*.

[30] Repko A, Nižňanský D, Poltierová-Vejpravová J (2011). A study of oleic acid-based hydrothermal preparation of CoFe_2_O_4_ nanoparticles. *Journal of Nanoparticle Research*.

[31] Massart R, Roger J, Cabuil V (1995). New trends in chemistry of magnetic colloids: polar and non polar magnetic fluids, emulsions, capsules and vesicles. *Brazilian Journal of Physics*.

[32] Faraji M, Yamini Y, Rezaee M (2010). Magnetic nanoparticles: synthesis, stabilization, functionalization, characterization, and applications. *Journal of the Iranian Chemical Society*.

[33] Pauline S, Amaliya AP (2011). Synthesis and characterization of highly monodispersive CoFe_2_O_4_ magnetic nanoparticles by hydrothermal chemical route. *Archives of Applied Science Research*.

[34] Yue-Jian C, Juan T, Fei X (2010). Synthesis, self-assembly, and characterization of PEG-coated iron oxide nanoparticles as potential MRI contrast agent. *Drug Development and Industrial Pharmacy*.

[35] Gao F, Cai Y, Zhou J (2010). Pullulan acetate coated magnetite nanoparticles for hyper-thermia: preparation, characterization and in vitro experiments. *Nano Research*.

[36] Liong M, Lu J, Kovochich M (2008). Multifunctional inorganic nanoparticles for imaging, targeting, and drug delivery. *ACS Nano*.

[37] Tartaj P, Del Puerto Morales M, Veintemillas-Verdaguer S, González-Carreño T, Serna CJ (2003). The preparation of magnetic nanoparticles for applications in biomedicine. *Journal of Physics D*.

[38] Mehta RV, Desai R, Bhatt P, Upadhyay RV (2006). Synthesis and characterization of certain nanomagnetic particles coated with citrate and dextran molecules. *Indian Journal of Pure and Applied Physics*.

[39] Purushotham S, Chang PEJ, Rumpel H (2009). Thermoresponsive core-shell magnetic nanoparticles for combined modalities of cancer therapy. *Nanotechnology*.

[40] Morales MA, Jain TK, Labhasetwar V, Leslie-Pelecky DL (2005). Magnetic studies of iron oxide nanoparticles coated with oleic acid and pluronic block copolymer. *Journal of Applied Physics*.

[41] Yang X, Chen Y, Yuan R (2008). Folate-encoded and Fe_3_O_4_-loaded polymeric micelles for dual targeting of cancer cells. *Polymer*.

[42] Yang X, Deng W, Fu L (2008). Folate-functionalized polymeric micelles for tumor targeted delivery of a potent multidrug-resistance modulator FG020326. *Journal of Biomedical Materials Research A*.

[43] Hayama A, Yamamoto T, Yokoyama M, Kawano K, Hattori Y, Maitani Y (2008). Polymeric micelles modified by folate-peg-lipid for targeted drug delivery to cancer cells in vitro. *Journal of Nanoscience and Nanotechnology*.

[44] Zhang J, Misra RDK (2007). Magnetic drug-targeting carrier encapsulated with thermosensitive smart polymer: core-shell nanoparticle carrier and drug release response. *Acta Biomaterialia*.

[45] Zhu L, Ma J, Jia N, Zhao Y, Shen H (2009). Chitosan-coated magnetic nanoparticles as carriers of 5-fluorouracil: preparation, characterization and cytotoxicity studies. *Colloids and Surfaces B*.

[46] Zhang L, He R, Gu H-C (2006). Oleic acid coating on the monodisperse magnetite nanoparticles. *Applied Surface Science*.

[47] Fan L, Zhang Y, Luo C, Lu F, Qiu H, Sun M (2012). Synthesis and characterization of magnetic *β*-cyclodextrin-chitosan nanoparticles as nano-adsorbents for removal of methyl blue. *International Journal of Biological Macromolecules*.

[48] Tao Y-T (1993). Structural comparison of self-assembled monolayers of n-alkanoic acids on the surfaces of silver, copper, and aluminum. *Journal of the American Chemical Society*.

[49] Gonzales M, Krishnan KM (2007). Phase transfer of highly monodisperse iron oxide nanocrystals with Pluronic F127 for biomedical applications. *Journal of Magnetism and Magnetic Materials*.

[50] Zhao SY, Lee D-G, Kim C-W, Cha H-G, Kim Y-H, Kang Y-S (2006). Synthesis of magnetic nanoparticles of Fe_3_O_4_ and CoFe_2_O_4_ and their surface modification by surfactant adsorption. *Bulletin of the Korean Chemical Society*.

[51] Chandrasekharan P, Maity D, Yong CX, Chuang K-H, Ding J, Feng S-S (2011). Vitamin E (d-alpha-tocopheryl-co-poly(ethylene glycol) 1000 succinate) micelles-superparamagnetic iron oxide nanoparticles for enhanced thermotherapy and MRI. *Biomaterials*.

[52] Yallapu MM, Othman SF, Curtis ET, Bauer NA, Chauhan N, Kumar D (2012). Curcumin-loaded magnetic nanoparticles for breast cancer therapeutics and imaging applications. *International Journal of Nanomedicine*.

[53] Sun C, Veiseh O, Gunn J (2008). In vivo MRI detection of gliomas by chlorotoxin-conjugated superparamagnetic nanoprobes. *Small*.

[54] Meier R, Henning TD, Boddington S (2010). Breast cancers: MR imaging of folate-receptor expression with the folate-specific nanoparticle P1133. *Radiology*.

[55] Shakeri-Zadeh A, Mansoori GA, Hashemian AR, Eshghi H, Sazgarnia A, Montazerabadi AR (2010). Cancerous cells targeting and destruction using folate conjugated gold nanoparticles. *Dynamic Biochemistry Process Biotechnology and Molecular Biology*.

